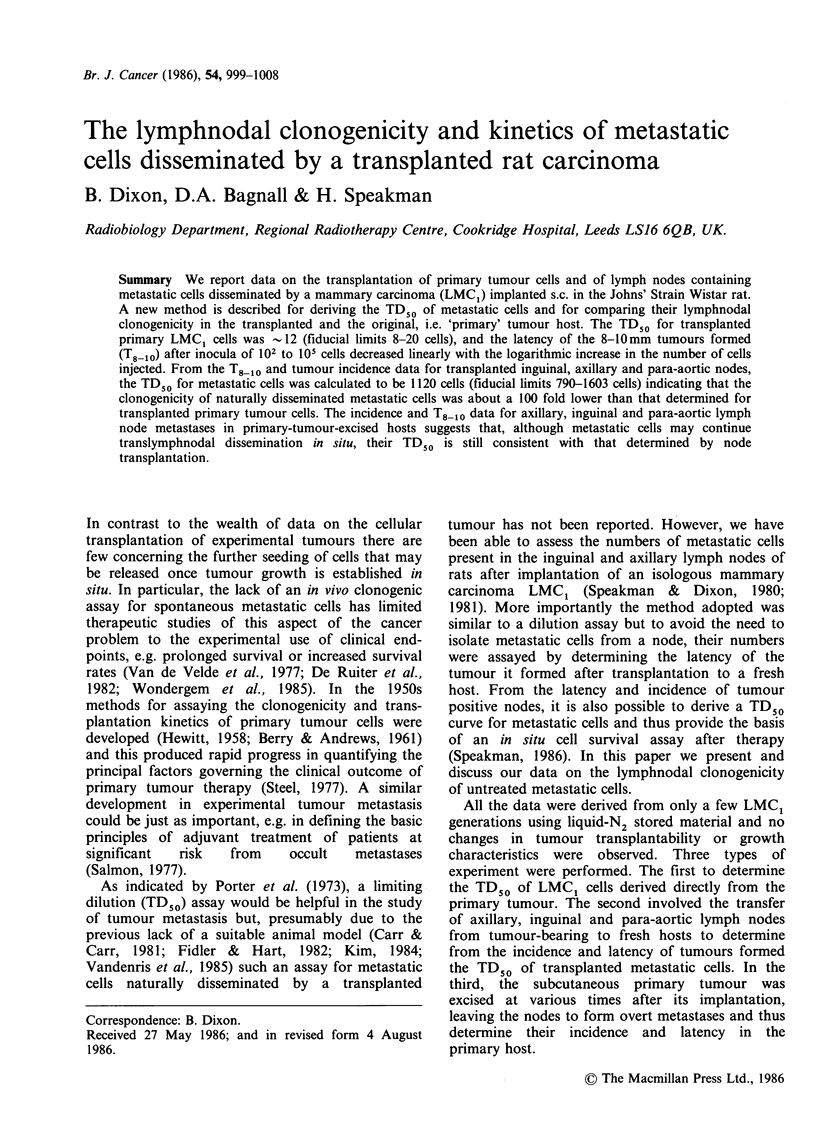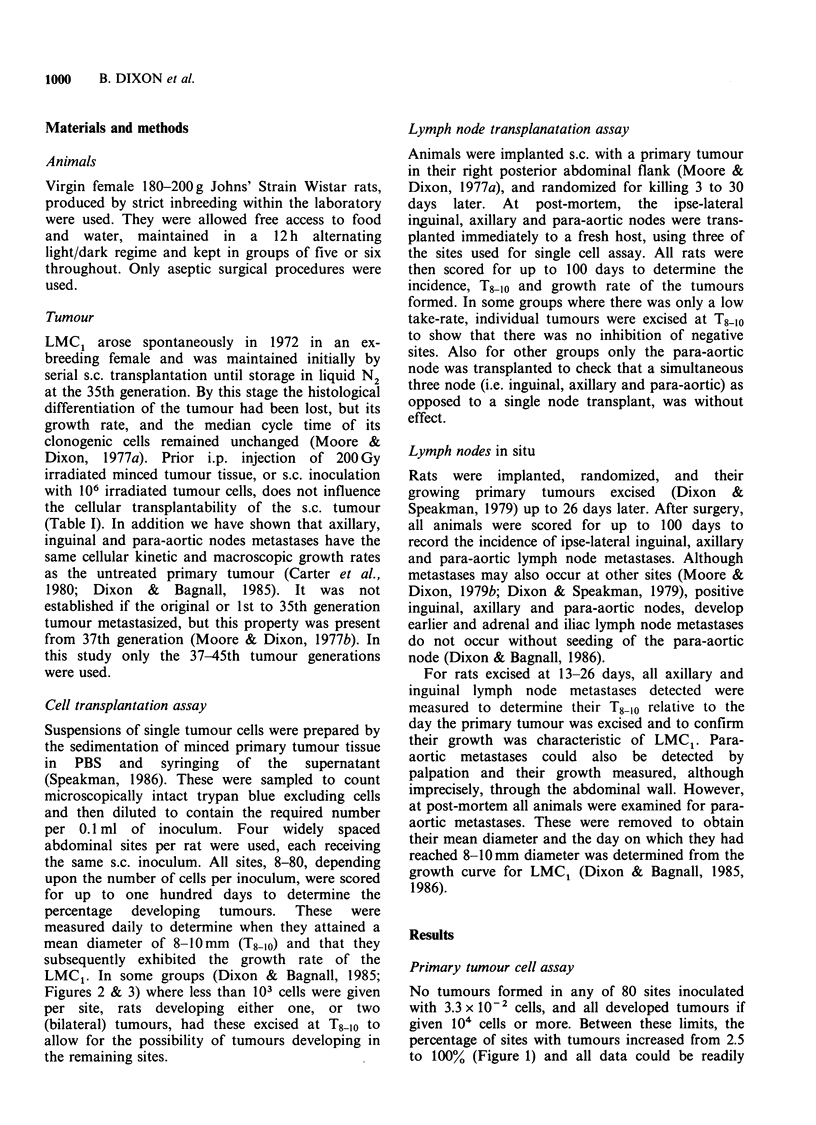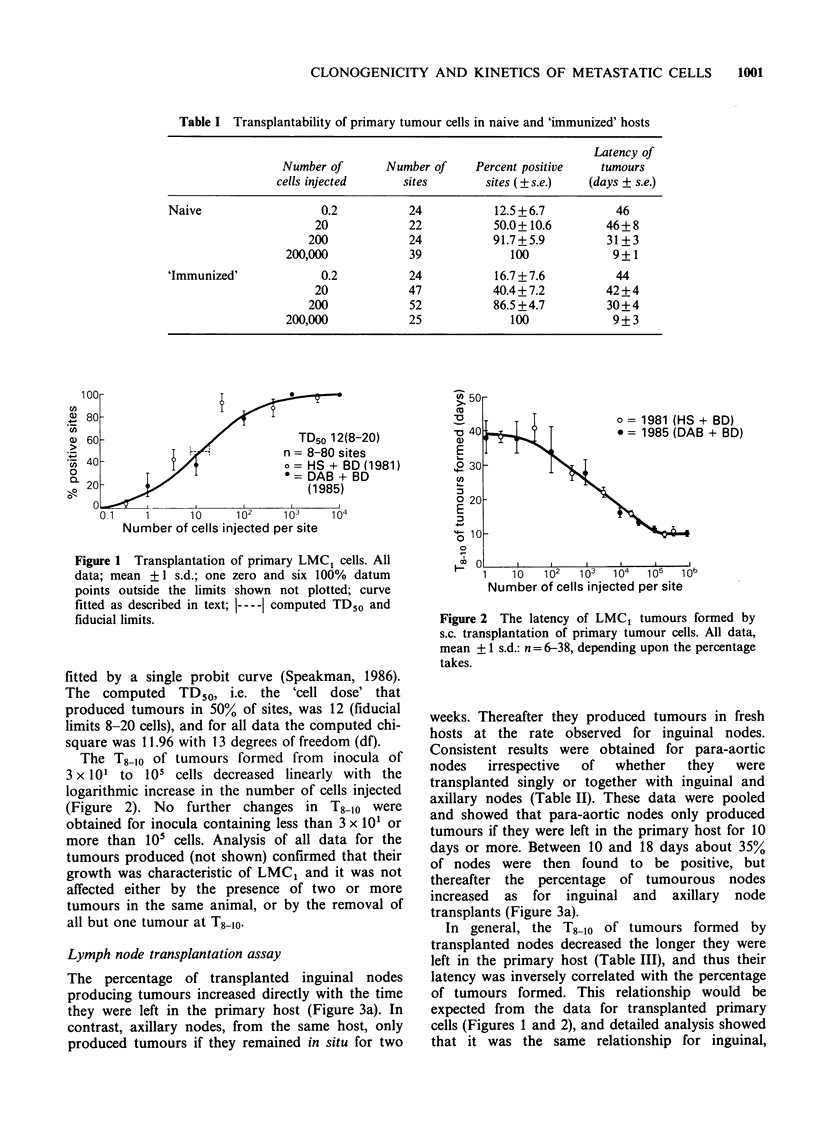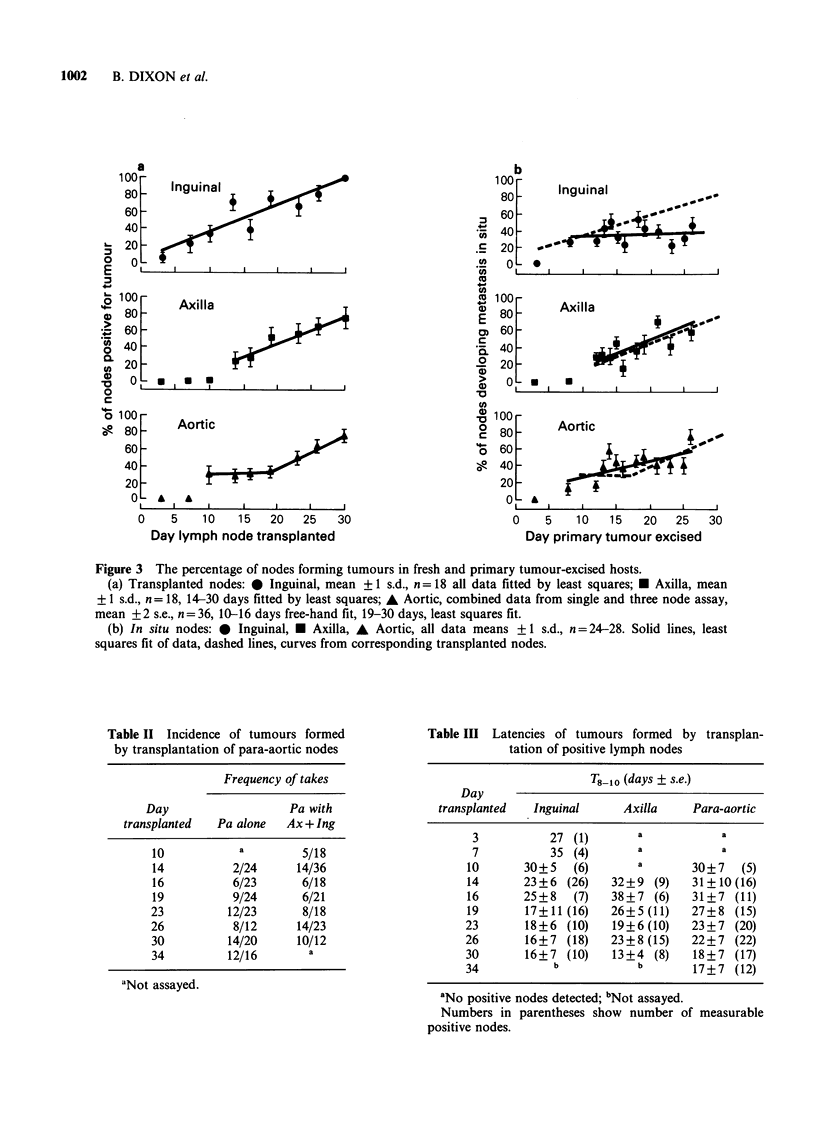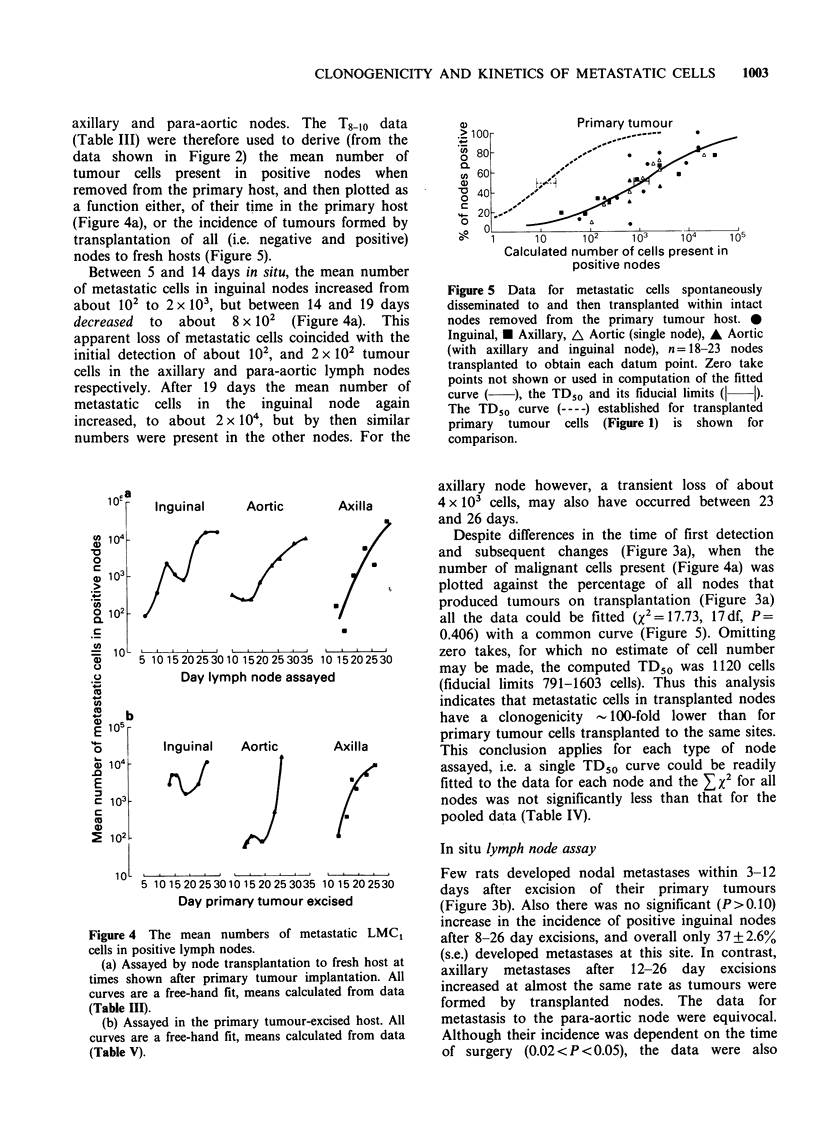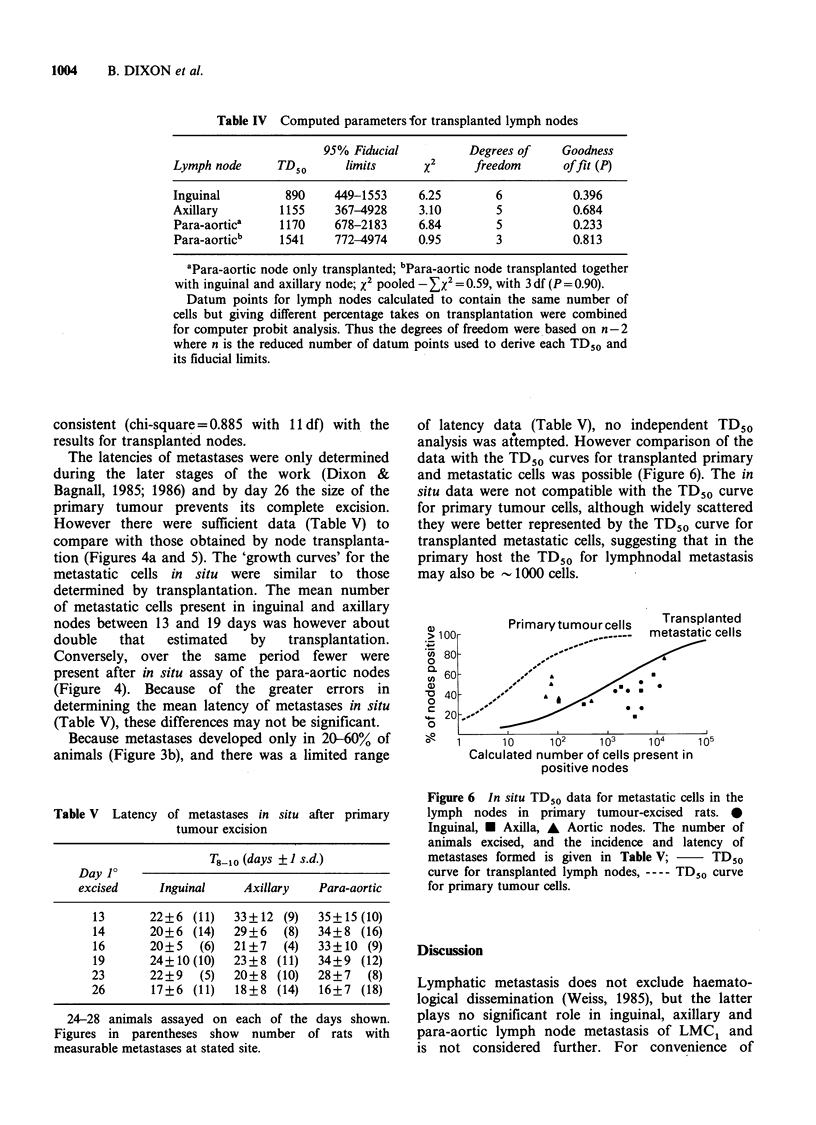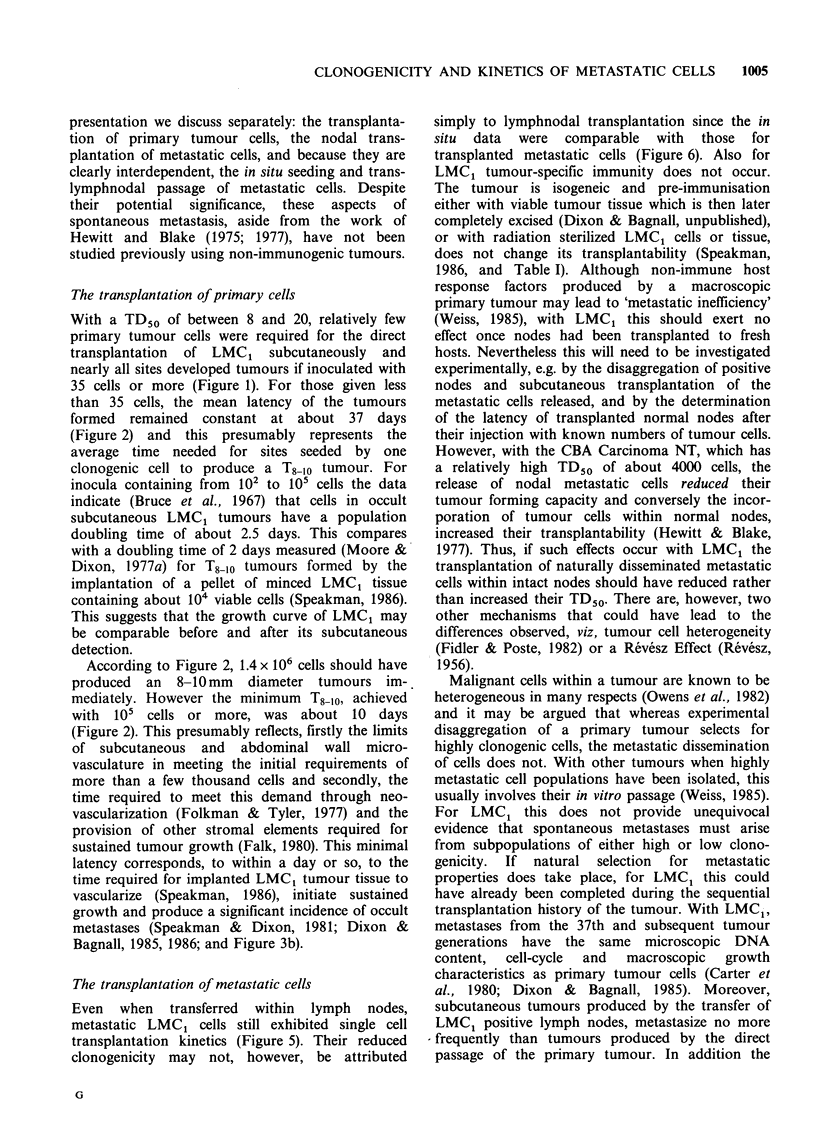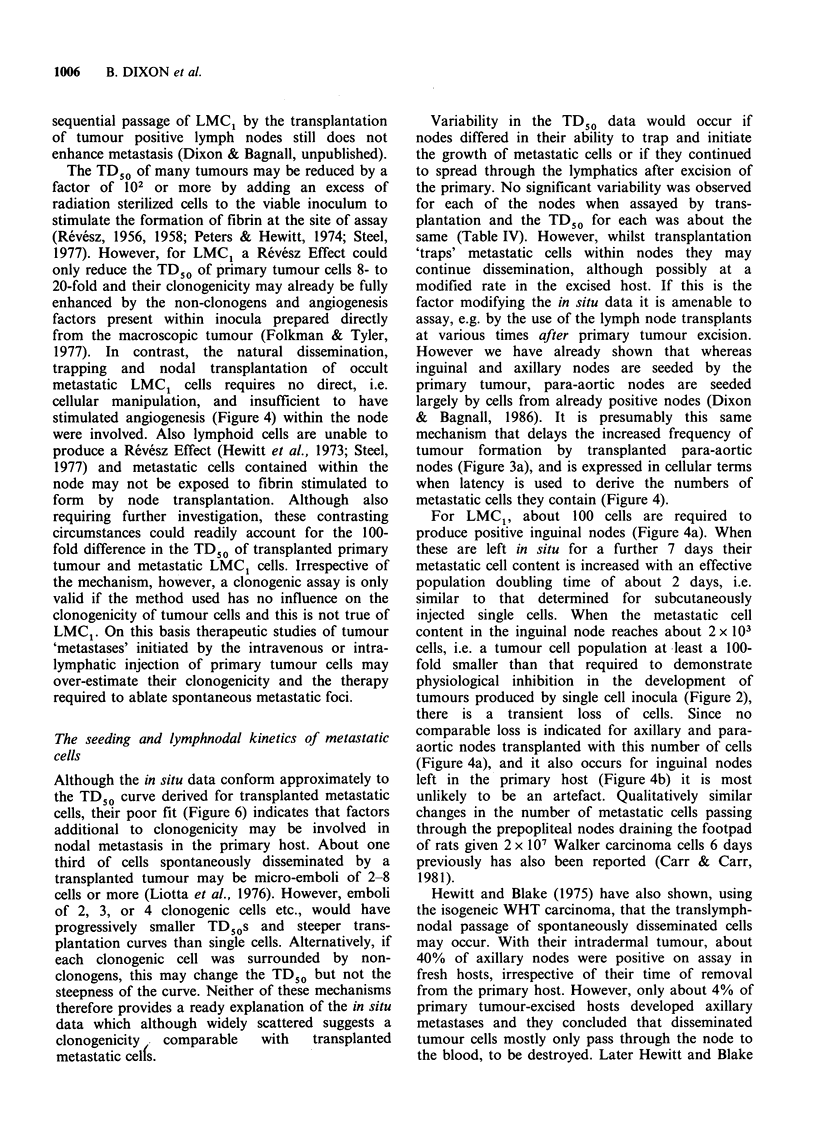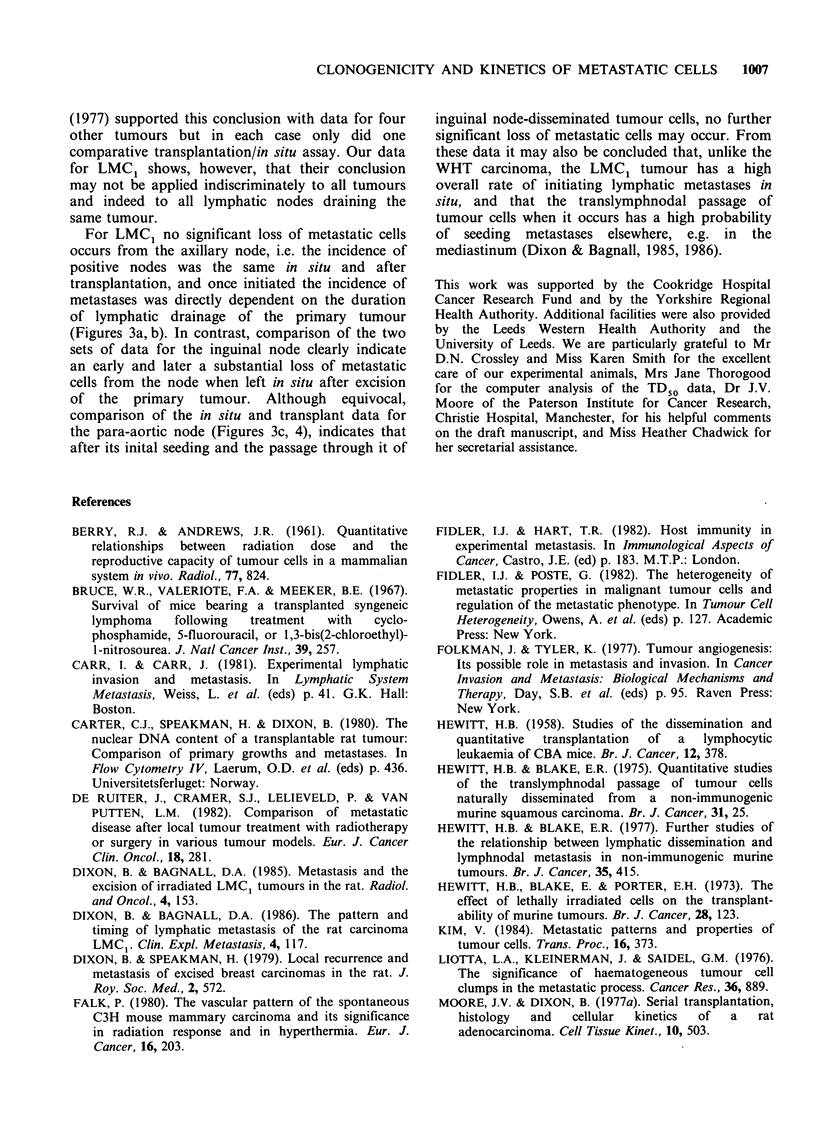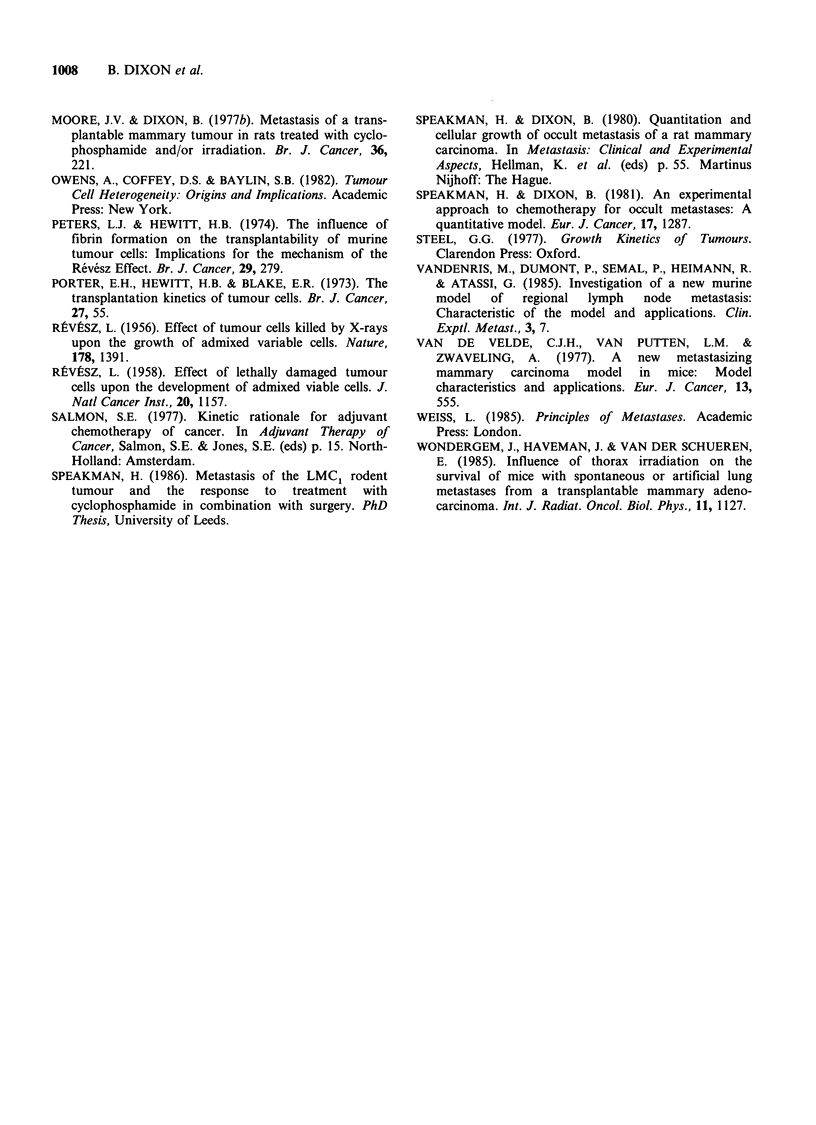# The lymphnodal clonogenicity and kinetics of metastatic cells disseminated by a transplanted rat carcinoma.

**DOI:** 10.1038/bjc.1986.272

**Published:** 1986-12

**Authors:** B. Dixon, D. A. Bagnall, H. Speakman

## Abstract

We report data on the transplantation of primary tumour cells and of lymph nodes containing metastatic cells disseminated by a mammary carcinoma (LMC1) implanted s.c. in the Johns' Strain Wistar rat. A new method is described for deriving the TD50 of metastatic cells and for comparing their lymphnodal clonogenicity in the transplanted and the original, i.e. 'primary' tumour host. The TD50 for transplanted primary LMC1 cells was approximately 12 (fiducial limits 8-20 cells), and the latency of the 8-10mm tumours formed (T8-10) after inocula of 10(2) to 10(5) cells decreased linearly with the logarithmic increase in the number of cells injected. From the T8-10 and tumour incidence data for transplanted inguinal, axillary and para-aortic nodes, the TD50 for metastatic cells was calculated to be 1120 cells (fiducial limits 790-1603 cells) indicating that the clonogenicity of naturally disseminated metastatic cells was about a 100 fold lower than that determined for transplanted primary tumour cells. The incidence and T8-10 data for axillary, inguinal and para-aortic lymph node metastases in primary-tumour-excised hosts suggests that, although metastatic cells may continue translymphnodal dissemination in situ, their TD50 is still consistent with that determined by node transplantation.


*Br. J. Cancer* (1986), **54**, 999–1008

### The lymphnodal clonogenicity and kinetics of metastatic cells disseminated by a transplanted rat carcinoma

B. Dixon, D.A. Bagnall & H. Speakman

*Radiobiology Department, Regional Radiotherapy Centre, Cookridge Hospital, Leeds LS16 6QB, UK.*

**Summary** We report data on the transplantation of primary tumour cells and of lymph nodes containing metastatic cells disseminated by a mammary carcinoma ($LMC_1$) implanted s.c. in the Johns' Strain Wistar rat. A new method is described for deriving the $TD_{50}$ of metastatic cells and for comparing their lymphnodal clonogenicity in the transplanted and the original, i.e. 'primary' tumour host. The $TD_{50}$ for transplanted primary $LMC_1$ cells was ~12 (fiducial limits 8–20 cells), and the latency of the 8–10 mm tumours formed ($T_{8-10}$) after inocula of $10^2$ to $10^5$ cells decreased linearly with the logarithmic increase in the number of cells injected. From the $T_{8-10}$ and tumour incidence data for transplanted inguinal, axillary and para-aortic nodes, the $TD_{50}$ for metastatic cells was calculated to be 1120 cells (fiducial limits 790–1603 cells) indicating that the clonogenicity of naturally disseminated metastatic cells was about a 100 fold lower than that determined for transplanted primary tumour cells. The incidence and $T_{8-10}$ data for axillary, inguinal and para-aortic lymph node metastases in primary-tumour-excised hosts suggests that, although metastatic cells may continue translymphnodal dissemination *in situ*, their $TD_{50}$ is still consistent with that determined by node transplantation.

In contrast to the wealth of data on the cellular transplantation of experimental tumours there are few concerning the further seeding of cells that may be released once tumour growth is established *in situ*. In particular, the lack of an *in vivo* clonogenic assay for spontaneous metastatic cells has limited therapeutic studies of this aspect of the cancer problem to the experimental use of clinical end-points, e.g. prolonged survival or increased survival rates (Van de Velde *et al.*, 1977; De Ruiter *et al.*, 1982; Wondergem *et al.*, 1985). In the 1950s methods for assaying the clonogenicity and trans-plantation kinetics of primary tumour cells were developed (Hewitt, 1958; Berry & Andrews, 1961) and this produced rapid progress in quantifying the principal factors governing the clinical outcome of primary tumour therapy (Steel, 1977). A similar development in experimental tumour metastasis could be just as important, e.g. in defining the basic principles of adjuvant treatment of patients at significant risk from occult metastases (Salmon, 1977).

As indicated by Porter *et al.* (1973), a limiting dilution ($TD_{50}$) assay would be helpful in the study of tumour metastasis but, presumably due to the previous lack of a suitable animal model (Carr & Carr, 1981; Fidler & Hart, 1982; Kim, 1984; Vandenris *et al.*, 1985) such an assay for metastatic cells naturally disseminated by a transplanted tumour has not been reported. However, we have been able to assess the numbers of metastatic cells present in the inguinal and axillary lymph nodes of rats after implantation of an isologous mammary carcinoma $LMC_1$ (Speakman & Dixon, 1980; 1981). More importantly the method adopted was similar to a dilution assay but to avoid the need to isolate metastatic cells from a node, their numbers were assayed by determining the latency of the tumour it formed after transplantation to a fresh host. From the latency and incidence of tumour positive nodes, it is also possible to derive a $TD_{50}$ curve for metastatic cells and thus provide the basis of an *in situ* cell survival assay after therapy (Speakman, 1986). In this paper we present and discuss our data on the lymphnodal clonogenicity of untreated metastatic cells.

All the data were derived from only a few $LMC_1$ generations using liquid-$N_2$ stored material and no changes in tumour transplantability or growth characteristics were observed. Three types of experiment were performed. The first to determine the $TD_{50}$ of $LMC_1$ cells derived directly from the primary tumour. The second involved the transfer of axillary, inguinal and para-aortic lymph nodes from tumour-bearing to fresh hosts to determine from the incidence and latency of tumours formed the $TD_{50}$ of transplanted metastatic cells. In the third, the subcutaneous primary tumour was excised at various times after its implantation, leaving the nodes to form overt metastases and thus determine their incidence and latency in the primary host.

Correspondence: B. Dixon.
Received 27 May 1986; and in revised form 4 August 1986.

© The Macmillan Press Ltd., 1986

### Materials and methods

### *Animals*

Virgin female 180–200 g Johns' Strain Wistar rats, produced by strict inbreeding within the laboratory were used. They were allowed free access to food and water, maintained in a 12 h alternating light/dark regime and kept in groups of five or six throughout. Only aseptic surgical procedures were used.

### *Tumour*

$LMC_1$ arose spontaneously in 1972 in an ex-breeding female and was maintained initially by serial s.c. transplantation until storage in liquid $N_2$ at the 35th generation. By this stage the histological differentiation of the tumour had been lost, but its growth rate, and the median cycle time of its clonogenic cells remained unchanged (Moore & Dixon, 1977*a*). Prior i.p. injection of 200 Gy irradiated minced tumour tissue, or s.c. inoculation with $10^6$ irradiated tumour cells, does not influence the cellular transplantability of the s.c. tumour (Table I). In addition we have shown that axillary, inguinal and para-aortic nodes metastases have the same cellular kinetic and macroscopic growth rates as the untreated primary tumour (Carter *et al.*, 1980; Dixon & Bagnall, 1985). It was not established if the original or 1st to 35th generation tumour metastasized, but this property was present from 37th generation (Moore & Dixon, 1977*b*). In this study only the 37–45th tumour generations were used.

### *Cell transplantation assay*

Suspensions of single tumour cells were prepared by the sedimentation of minced primary tumour tissue in PBS and syringing of the supernatant (Speakman, 1986). These were sampled to count microscopically intact trypan blue excluding cells and then diluted to contain the required number per 0.1 ml of inoculum. Four widely spaced abdominal sites per rat were used, each receiving the same s.c. inoculum. All sites, 8–80, depending upon the number of cells per inoculum, were scored for up to one hundred days to determine the percentage developing tumours. These were measured daily to determine when they attained a mean diameter of 8–10 mm ($T_{8-10}$) and that they subsequently exhibited the growth rate of the $LMC_1$. In some groups (Dixon & Bagnall, 1985; Figures 2 & 3) where less than $10^3$ cells were given per site, rats developing either one, or two (bilateral) tumours, had these excised at $T_{8-10}$ to allow for the possibility of tumours developing in the remaining sites.

### *Lymph node transplanatation assay*

Animals were implanted s.c. with a primary tumour in their right posterior abdominal flank (Moore & Dixon, 1977*a*), and randomized for killing 3 to 30 days later. At post-mortem, the ipse-lateral inguinal, axillary and para-aortic nodes were transplanted immediately to a fresh host, using three of the sites used for single cell assay. All rats were then scored for up to 100 days to determine the incidence, $T_{8-10}$ and growth rate of the tumours formed. In some groups where there was only a low take-rate, individual tumours were excised at $T_{8-10}$ to show that there was no inhibition of negative sites. Also for other groups only the para-aortic node was transplanted to check that a simultaneous three node (i.e. inguinal, axillary and para-aortic) as opposed to a single node transplant, was without effect.

### *Lymph nodes* in situ

Rats were implanted, randomized, and their growing primary tumours excised (Dixon & Speakman, 1979) up to 26 days later. After surgery, all animals were scored for up to 100 days to record the incidence of ipse-lateral inguinal, axillary and para-aortic lymph node metastases. Although metastases may also occur at other sites (Moore & Dixon, 1979*b*; Dixon & Speakman, 1979), positive inguinal, axillary and para-aortic nodes, develop earlier and adrenal and iliac lymph node metastases do not occur without seeding of the para-aortic node (Dixon & Bagnall, 1986).

For rats excised at 13–26 days, all axillary and inguinal lymph node metastases detected were measured to determine their $T_{8-10}$ relative to the day the primary tumour was excised and to confirm their growth was characteristic of $LMC_1$. Para-aortic metastases could also be detected by palpation and their growth measured, although imprecisely, through the abdominal wall. However, at post-mortem all animals were examined for para-aortic metastases. These were removed to obtain their mean diameter and the day on which they had reached 8–10 mm diameter was determined from the growth curve for $LMC_1$ (Dixon & Bagnall, 1985, 1986).

### Results

### *Primary tumour cell assay*

No tumours formed in any of 80 sites inoculated with $3.3 \times 10^{-2}$ cells, and all developed tumours if given $10^4$ cells or more. Between these limits, the percentage of sites with tumours increased from 2.5 to 100% (Figure 1) and all data could be readily

**Table I** Transplantability of primary tumour cells in naive and 'immunized' hosts

| | *Number of cells injected* | *Number of sites* | *Percent positive sites (±s.e.)* | *Latency of tumours (days ± s.e.)* |
|---|---|---|---|---|
| Naive | 0.2 | 24 | 12.5±6.7 | 46 |
| | 20 | 22 | 50.0±10.6 | 46±8 |
| | 200 | 24 | 91.7±5.9 | 31±3 |
| | 200,000 | 39 | 100 | 9±1 |
| 'Immunized' | 0.2 | 24 | 16.7±7.6 | 44 |
| | 20 | 47 | 40.4±7.2 | 42±4 |
| | 200 | 52 | 86.5±4.7 | 30±4 |
| | 200,000 | 25 | 100 | 9±3 |

**Figure 1** Transplantation of primary $LMC_1$ cells. All data; mean ±1 s.d.; one zero and six 100% datum points outside the limits shown not plotted; curve fitted as described in text; |---| computed $TD_{50}$ and fiducial limits.

**Figure 2** The latency of $LMC_1$ tumours formed by s.c. transplantation of primary tumour cells. All data, mean ±1 s.d.: $n = 6–38$, depending upon the percentage takes.

fitted by a single probit curve (Speakman, 1986). The computed $TD_{50}$, i.e. the 'cell dose' that produced tumours in 50% of sites, was 12 (fiducial limits 8–20 cells), and for all data the computed chi-square was 11.96 with 13 degrees of freedom (df).

The $T_{8-10}$ of tumours formed from inocula of $3 \times 10^1$ to $10^5$ cells decreased linearly with the logarithmic increase in the number of cells injected (Figure 2). No further changes in $T_{8-10}$ were obtained for inocula containing less than $3 \times 10^1$ or more than $10^5$ cells. Analysis of all data for the tumours produced (not shown) confirmed that their growth was characteristic of $LMC_1$ and it was not affected either by the presence of two or more tumours in the same animal, or by the removal of all but one tumour at $T_{8-10}$.

### *Lymph node transplantation assay*

The percentage of transplanted inguinal nodes producing tumours increased directly with the time they were left in the primary host (Figure 3a). In contrast, axillary nodes, from the same host, only produced tumours if they remained *in situ* for two weeks. Thereafter they produced tumours in fresh hosts at the rate observed for inguinal nodes. Consistent results were obtained for para-aortic nodes irrespective of whether they were transplanted singly or together with inguinal and axillary nodes (Table II). These data were pooled and showed that para-aortic nodes only produced tumours if they were left in the primary host for 10 days or more. Between 10 and 18 days about 35% of nodes were then found to be positive, but thereafter the percentage of tumourous nodes increased as for inguinal and axillary node transplants (Figure 3a).

In general, the $T_{8-10}$ of tumours formed by transplanted nodes decreased the longer they were left in the primary host (Table III), and thus their latency was inversely correlated with the percentage of tumours formed. This relationship would be expected from the data for transplanted primary cells (Figures 1 and 2), and detailed analysis showed that it was the same relationship for inguinal,

**Figure 3** The percentage of nodes forming tumours in fresh and primary tumour-excised hosts.

(a) Transplanted nodes: ● Inguinal, mean ±1 s.d., $n=18$ all data fitted by least squares; ■ Axilla, mean ±1 s.d., $n=18$, 14–30 days fitted by least squares; ▲ Aortic, combined data from single and three node assay, mean ±2 s.e., $n=36$, 10–16 days free-hand fit, 19–30 days, least squares fit.

(b) *In situ* nodes: ● Inguinal, ■ Axilla, ▲ Aortic, all data means ±1 s.d., $n=24$–28. Solid lines, least squares fit of data, dashed lines, curves from corresponding transplanted nodes.

**Table II** Incidence of tumours formed by transplantation of para-aortic nodes

| | *Frequency of takes* | |
|---|---|---|
| *Day transplanted* | *Pa alone* | *Pa with Ax+Ing* |
| 10 | [a] | 5/18 |
| 14 | 2/24 | 14/36 |
| 16 | 6/23 | 6/18 |
| 19 | 9/24 | 6/21 |
| 23 | 12/23 | 8/18 |
| 26 | 8/12 | 14/23 |
| 30 | 14/20 | 10/12 |
| 34 | 12/16 | [a] |

[a]Not assayed.

**Table III** Latencies of tumours formed by transplantation of positive lymph nodes

| *Day transplanted* | $T_{8-10}$ (*days* ± *s.e.*) | | |
|---|---|---|---|
| | *Inguinal* | *Axilla* | *Para-aortic* |
| 3 | 27 (1) | [a] | [a] |
| 7 | 35 (4) | [a] | [a] |
| 10 | 30±5 (6) | [a] | 30±7 (5) |
| 14 | 23±6 (26) | 32±9 (9) | 31±10 (16) |
| 16 | 25±8 (7) | 38±7 (6) | 31±7 (11) |
| 19 | 17±11 (16) | 26±5 (11) | 27±8 (15) |
| 23 | 18±6 (10) | 19±6 (10) | 23±7 (20) |
| 26 | 16±7 (18) | 23±8 (15) | 22±7 (22) |
| 30 | 16±7 (10) | 13±4 (8) | 18±7 (17) |
| 34 | [b] | [b] | 17±7 (12) |

[a]No positive nodes detected; [b]Not assayed.

Numbers in parentheses show number of measurable positive nodes.

axillary and para-aortic nodes. The $T_{8\text{-}10}$ data (Table III) were therefore used to derive (from the data shown in Figure 2) the mean number of tumour cells present in positive nodes when removed from the primary host, and then plotted as a function either, of their time in the primary host (Figure 4a), or the incidence of tumours formed by transplantation of all (i.e. negative and positive) nodes to fresh hosts (Figure 5).

Between 5 and 14 days *in situ*, the mean number of metastatic cells in inguinal nodes increased from about $10^2$ to $2\times10^3$, but between 14 and 19 days *decreased* to about $8\times10^2$ (Figure 4a). This apparent loss of metastatic cells coincided with the initial detection of about $10^2$, and $2\times10^2$ tumour cells in the axillary and para-aortic lymph nodes respectively. After 19 days the mean number of metastatic cells in the inguinal node again increased, to about $2\times10^4$, but by then similar numbers were present in the other nodes. For the axillary node however, a transient loss of about $4\times10^3$ cells, may also have occurred between 23 and 26 days.

**Figure 5** Data for metastatic cells spontaneously disseminated to and then transplanted within intact nodes removed from the primary tumour host. ● Inguinal, ■ Axillary, △ Aortic (single node), ▲ Aortic (with axillary and inguinal node), $n=18$–23 nodes transplanted to obtain each datum point. Zero take points not shown or used in computation of the fitted curve (——), the $TD_{50}$ and its fiducial limits (|——|). The $TD_{50}$ curve (----) established for transplanted primary tumour cells (**Figure 1**) is shown for comparison.

**Figure 4** The mean numbers of metastatic $LMC_1$ cells in positive lymph nodes.

(a) Assayed by node transplantation to fresh host at times shown after primary tumour implantation. All curves are a free-hand fit, means calculated from data (**Table III**).

(b) Assayed in the primary tumour-excised host. All curves are a free-hand fit, means calculated from data (**Table V**).

Despite differences in the time of first detection and subsequent changes (Figure 3a), when the number of malignant cells present (Figure 4a) was plotted against the percentage of all nodes that produced tumours on transplantation (Figure 3a) all the data could be fitted ($\chi^2=17.73$, 17 df, $P=0.406$) with a common curve (Figure 5). Omitting zero takes, for which no estimate of cell number may be made, the computed $TD_{50}$ was 1120 cells (fiducial limits 791–1603 cells). Thus this analysis indicates that metastatic cells in transplanted nodes have a clonogenicity ~100-fold lower than for primary tumour cells transplanted to the same sites. This conclusion applies for each type of node assayed, i.e. a single $TD_{50}$ curve could be readily fitted to the data for each node and the $\sum\chi^2$ for all nodes was not significantly less than that for the pooled data (Table IV).

### In situ *lymph node assay*

Few rats developed nodal metastases within 3–12 days after excision of their primary tumours (Figure 3b). Also there was no significant ($P>0.10$) increase in the incidence of positive inguinal nodes after 8–26 day excisions, and overall only $37\pm2.6\%$ (s.e.) developed metastases at this site. In contrast, axillary metastases after 12–26 day excisions increased at almost the same rate as tumours were formed by transplanted nodes. The data for metastasis to the para-aortic node were equivocal. Although their incidence was dependent on the time of surgery ($0.02<P<0.05$), the data were also

Table IV Computed parameters for transplanted lymph nodes

| *Lymph node* | $TD_{50}$ | *95% Fiducial limits* | $\chi^2$ | *Degrees of freedom* | *Goodness of fit (P)* |
|---|---|---|---|---|---|
| Inguinal | 890 | 449–1553 | 6.25 | 6 | 0.396 |
| Axillary | 1155 | 367–4928 | 3.10 | 5 | 0.684 |
| Para-aortic[a] | 1170 | 678–2183 | 6.84 | 5 | 0.233 |
| Para-aortic[b] | 1541 | 772–4974 | 0.95 | 3 | 0.813 |

[a]Para-aortic node only transplanted; [b]Para-aortic node transplanted together with inguinal and axillary node; $\chi^2$ pooled $-\sum\chi^2=0.59$, with 3 df ($P=0.90$).

Datum points for lymph nodes calculated to contain the same number of cells but giving different percentage takes on transplantation were combined for computer probit analysis. Thus the degrees of freedom were based on $n-2$ where $n$ is the reduced number of datum points used to derive each $TD_{50}$ and its fiducial limits.

consistent (chi-square = 0.885 with 11 df) with the results for transplanted nodes.

The latencies of metastases were only determined during the later stages of the work (Dixon & Bagnall, 1985; 1986) and by day 26 the size of the primary tumour prevents its complete excision. However there were sufficient data (Table V) to compare with those obtained by node transplantation (Figures 4a and 5). The 'growth curves' for the metastatic cells *in situ* were similar to those determined by transplantation. The mean number of metastatic cells present in inguinal and axillary nodes between 13 and 19 days was however about double that estimated by transplantation. Conversely, over the same period fewer were present after *in situ* assay of the para-aortic nodes (Figure 4). Because of the greater errors in determining the mean latency of metastases *in situ* (Table V), these differences may not be significant.

Because metastases developed only in 20–60% of animals (Figure 3b), and there was a limited range of latency data (Table V), no independent $TD_{50}$ analysis was attempted. However comparison of the data with the $TD_{50}$ curves for transplanted primary and metastatic cells was possible (Figure 6). The *in situ* data were not compatible with the $TD_{50}$ curve for primary tumour cells, although widely scattered they were better represented by the $TD_{50}$ curve for transplanted metastatic cells, suggesting that in the primary host the $TD_{50}$ for lymphnodal metastasis may also be $\sim$1000 cells.

Table V Latency of metastases *in situ* after primary tumour excision

| *Day 1° excised* | $T_{8-10}$ *(days ±1 s.d.)* Inguinal | Axillary | Para-aortic |
|---|---|---|---|
| 13 | 22±6 (11) | 33±12 (9) | 35±15 (10) |
| 14 | 20±6 (14) | 29±6 (8) | 34±8 (16) |
| 16 | 20±5 (6) | 21±7 (4) | 33±10 (9) |
| 19 | 24±10 (10) | 23±8 (11) | 34±9 (12) |
| 23 | 22±9 (5) | 20±8 (10) | 28±7 (8) |
| 26 | 17±6 (11) | 18±8 (14) | 16±7 (18) |

24–28 animals assayed on each of the days shown. Figures in parentheses show number of rats with measurable metastases at stated site.

**Figure 6** *In situ* $TD_{50}$ data for metastatic cells in the lymph nodes in primary tumour-excised rats. ● Inguinal, ■ Axilla, ▲ Aortic nodes. The number of animals excised, and the incidence and latency of metastases formed is given in **Table V**; —— $TD_{50}$ curve for transplanted lymph nodes, ---- $TD_{50}$ curve for primary tumour cells.

### Discussion

Lymphatic metastasis does not exclude haematological dissemination (Weiss, 1985), but the latter plays no significant role in inguinal, axillary and para-aortic lymph node metastasis of $LMC_1$ and is not considered further. For convenience of presentation we discuss separately: the transplantation of primary tumour cells, the nodal transplantation of metastatic cells, and because they are clearly interdependent, the *in situ* seeding and trans-lymphnodal passage of metastatic cells. Despite their potential significance, these aspects of spontaneous metastasis, aside from the work of Hewitt and Blake (1975; 1977), have not been studied previously using non-immunogenic tumours.

*The transplantation of primary cells*

With a $TD_{50}$ of between 8 and 20, relatively few primary tumour cells were required for the direct transplantation of $LMC_1$ subcutaneously and nearly all sites developed tumours if inoculated with 35 cells or more (Figure 1). For those given less than 35 cells, the mean latency of the tumours formed remained constant at about 37 days (Figure 2) and this presumably represents the average time needed for sites seeded by one clonogenic cell to produce a $T_{8-10}$ tumour. For inocula containing from $10^2$ to $10^5$ cells the data indicate (Bruce *et al.*, 1967) that cells in occult subcutaneous $LMC_1$ tumours have a population doubling time of about 2.5 days. This compares with a doubling time of 2 days measured (Moore & Dixon, 1977*a*) for $T_{8-10}$ tumours formed by the implantation of a pellet of minced $LMC_1$ tissue containing about $10^4$ viable cells (Speakman, 1986). This suggests that the growth curve of $LMC_1$ may be comparable before and after its subcutaneous detection.

According to Figure 2, $1.4 \times 10^6$ cells should have produced an 8–10 mm diameter tumours immediately. However the minimum $T_{8-10}$, achieved with $10^5$ cells or more, was about 10 days (Figure 2). This presumably reflects, firstly the limits of subcutaneous and abdominal wall microvasculature in meeting the initial requirements of more than a few thousand cells and secondly, the time required to meet this demand through neovascularization (Folkman & Tyler, 1977) and the provision of other stromal elements required for sustained tumour growth (Falk, 1980). This minimal latency corresponds, to within a day or so, to the time required for implanted $LMC_1$ tumour tissue to vascularize (Speakman, 1986), initiate sustained growth and produce a significant incidence of occult metastases (Speakman & Dixon, 1981; Dixon & Bagnall, 1985, 1986; and Figure 3b).

*The transplantation of metastatic cells*

Even when transferred within lymph nodes, metastatic $LMC_1$ cells still exhibited single cell transplantation kinetics (Figure 5). Their reduced clonogenicity may not, however, be attributed simply to lymphnodal transplantation since the *in situ* data were comparable with those for transplanted metastatic cells (Figure 6). Also for $LMC_1$ tumour-specific immunity does not occur. The tumour is isogeneic and pre-immunisation either with viable tumour tissue which is then later completely excised (Dixon & Bagnall, unpublished), or with radiation sterilized $LMC_1$ cells or tissue, does not change its transplantability (Speakman, 1986, and Table I). Although non-immune host response factors produced by a macroscopic primary tumour may lead to 'metastatic inefficiency' (Weiss, 1985), with $LMC_1$ this should exert no effect once nodes had been transplanted to fresh hosts. Nevertheless this will need to be investigated experimentally, e.g. by the disaggregation of positive nodes and subcutaneous transplantation of the metastatic cells released, and by the determination of the latency of transplanted normal nodes after their injection with known numbers of tumour cells. However, with the CBA Carcinoma NT, which has a relatively high $TD_{50}$ of about 4000 cells, the release of nodal metastatic cells *reduced* their tumour forming capacity and conversely the incorporation of tumour cells within normal nodes, increased their transplantability (Hewitt & Blake, 1977). Thus, if such effects occur with $LMC_1$ the transplantation of naturally disseminated metastatic cells within intact nodes should have reduced rather than increased their $TD_{50}$. There are, however, two other mechanisms that could have lead to the differences observed, *viz,* tumour cell heterogeneity (Fidler & Poste, 1982) or a Révész Effect (Révész, 1956).

Malignant cells within a tumour are known to be heterogeneous in many respects (Owens *et al.*, 1982) and it may be argued that whereas experimental disaggregation of a primary tumour selects for highly clonogenic cells, the metastatic dissemination of cells does not. With other tumours when highly metastatic cell populations have been isolated, this usually involves their *in vitro* passage (Weiss, 1985). For $LMC_1$ this does not provide unequivocal evidence that spontaneous metastases must arise from subpopulations of either high or low clonogenicity. If natural selection for metastatic properties does take place, for $LMC_1$ this could have already been completed during the sequential transplantation history of the tumour. With $LMC_1$, metastases from the 37th and subsequent tumour generations have the same microscopic DNA content, cell-cycle and macroscopic growth characteristics as primary tumour cells (Carter *et al.*, 1980; Dixon & Bagnall, 1985). Moreover, subcutaneous tumours produced by the transfer of $LMC_1$ positive lymph nodes, metastasize no more frequently than tumours produced by the direct passage of the primary tumour. In addition the sequential passage of $LMC_1$ by the transplantation of tumour positive lymph nodes still does not enhance metastasis (Dixon & Bagnall, unpublished).

The $TD_{50}$ of many tumours may be reduced by a factor of $10^2$ or more by adding an excess of radiation sterilized cells to the viable inoculum to stimulate the formation of fibrin at the site of assay (Révész, 1956, 1958; Peters & Hewitt, 1974; Steel, 1977). However, for $LMC_1$ a Révész Effect could only reduce the $TD_{50}$ of primary tumour cells 8- to 20-fold and their clonogenicity may already be fully enhanced by the non-clonogens and angiogenesis factors present within inocula prepared directly from the macroscopic tumour (Folkman & Tyler, 1977). In contrast, the natural dissemination, trapping and nodal transplantation of occult metastatic $LMC_1$ cells requires no direct, i.e. cellular manipulation, and insufficient to have stimulated angiogenesis (Figure 4) within the node were involved. Also lymphoid cells are unable to produce a Révész Effect (Hewitt *et al.*, 1973; Steel, 1977) and metastatic cells contained within the node may not be exposed to fibrin stimulated to form by node transplantation. Although also requiring further investigation, these contrasting circumstances could readily account for the 100-fold difference in the $TD_{50}$ of transplanted primary tumour and metastatic $LMC_1$ cells. Irrespective of the mechanism, however, a clonogenic assay is only valid if the method used has no influence on the clonogenicity of tumour cells and this is not true of $LMC_1$. On this basis therapeutic studies of tumour 'metastases' initiated by the intravenous or intra-lymphatic injection of primary tumour cells may over-estimate their clonogenicity and the therapy required to ablate spontaneous metastatic foci.

### *The seeding and lymphnodal kinetics of metastatic cells*

Although the *in situ* data conform approximately to the $TD_{50}$ curve derived for transplanted metastatic cells, their poor fit (Figure 6) indicates that factors additional to clonogenicity may be involved in nodal metastasis in the primary host. About one third of cells spontaneously disseminated by a transplanted tumour may be micro-emboli of 2–8 cells or more (Liotta *et al.*, 1976). However, emboli of 2, 3, or 4 clonogenic cells etc., would have progressively smaller $TD_{50}$s and steeper transplantation curves than single cells. Alternatively, if each clonogenic cell was surrounded by non-clonogens, this may change the $TD_{50}$ but not the steepness of the curve. Neither of these mechanisms therefore provides a ready explanation of the *in situ* data which although widely scattered suggests a clonogenicity comparable with transplanted metastatic cells.

Variability in the $TD_{50}$ data would occur if nodes differed in their ability to trap and initiate the growth of metastatic cells or if they continued to spread through the lymphatics after excision of the primary. No significant variability was observed for each of the nodes when assayed by transplantation and the $TD_{50}$ for each was about the same (Table IV). However, whilst transplantation 'traps' metastatic cells within nodes they may continue dissemination, although possibly at a modified rate in the excised host. If this is the factor modifying the *in situ* data it is amenable to assay, e.g. by the use of the lymph node transplants at various times *after* primary tumour excision. However we have already shown that whereas inguinal and axillary nodes are seeded by the primary tumour, para-aortic nodes are seeded largely by cells from already positive nodes (Dixon & Bagnall, 1986). It is presumably this same mechanism that delays the increased frequency of tumour formation by transplanted para-aortic nodes (Figure 3a), and is expressed in cellular terms when latency is used to derive the numbers of metastatic cells they contain (Figure 4).

For $LMC_1$, about 100 cells are required to produce positive inguinal nodes (Figure 4a). When these are left *in situ* for a further 7 days their metastatic cell content is increased with an effective population doubling time of about 2 days, i.e. similar to that determined for subcutaneously injected single cells. When the metastatic cell content in the inguinal node reaches about $2 \times 10^3$ cells, i.e. a tumour cell population at least a 100-fold smaller than that required to demonstrate physiological inhibition in the development of tumours produced by single cell inocula (Figure 2), there is a transient loss of cells. Since no comparable loss is indicated for axillary and para-aortic nodes transplanted with this number of cells (Figure 4a), and it also occurs for inguinal nodes left in the primary host (Figure 4b) it is most unlikely to be an artefact. Qualitatively similar changes in the number of metastatic cells passing through the prepopliteal nodes draining the footpad of rats given $2 \times 10^7$ Walker carcinoma cells 6 days previously has also been reported (Carr & Carr, 1981).

Hewitt and Blake (1975) have also shown, using the isogeneic WHT carcinoma, that the translymph-nodal passage of spontaneously disseminated cells may occur. With their intradermal tumour, about 40% of axillary nodes were positive on assay in fresh hosts, irrespective of their time of removal from the primary host. However, only about 4% of primary tumour-excised hosts developed axillary metastases and they concluded that disseminated tumour cells mostly only pass through the node to the blood, to be destroyed. Later Hewitt and Blake

(1977) supported this conclusion with data for four other tumours but in each case only did one comparative transplantation/*in situ* assay. Our data for $LMC_1$ shows, however, that their conclusion may not be applied indiscriminately to all tumours and indeed to all lymphatic nodes draining the same tumour.

For $LMC_1$ no significant loss of metastatic cells occurs from the axillary node, i.e. the incidence of positive nodes was the same *in situ* and after transplantation, and once initiated the incidence of metastases was directly dependent on the duration of lymphatic drainage of the primary tumour (Figures 3a, b). In contrast, comparison of the two sets of data for the inguinal node clearly indicate an early and later a substantial loss of metastatic cells from the node when left *in situ* after excision of the primary tumour. Although equivocal, comparison of the *in situ* and transplant data for the para-aortic node (Figures 3c, 4), indicates that after its inital seeding and the passage through it of inguinal node-disseminated tumour cells, no further significant loss of metastatic cells may occur. From these data it may also be concluded that, unlike the WHT carcinoma, the $LMC_1$ tumour has a high overall rate of initiating lymphatic metastases *in situ,* and that the translymphnodal passage of tumour cells when it occurs has a high probability of seeding metastases elsewhere, e.g. in the mediastinum (Dixon & Bagnall, 1985, 1986).

This work was supported by the Cookridge Hospital Cancer Research Fund and by the Yorkshire Regional Health Authority. Additional facilities were also provided by the Leeds Western Health Authority and the University of Leeds. We are particularly grateful to Mr D.N. Crossley and Miss Karen Smith for the excellent care of our experimental animals, Mrs Jane Thorogood for the computer analysis of the $TD_{50}$ data, Dr J.V. Moore of the Paterson Institute for Cancer Research, Christie Hospital, Manchester, for his helpful comments on the draft manuscript, and Miss Heather Chadwick for her secretarial assistance.

### References

BERRY, R.J. & ANDREWS, J.R. (1961). Quantitative relationships between radiation dose and the reproductive capacity of tumour cells in a mammalian system *in vivo. Radiol.*, **77**, 824.

BRUCE, W.R., VALERIOTE, F.A. & MEEKER, B.E. (1967). Survival of mice bearing a transplanted syngeneic lymphoma following treatment with cyclophosphamide, 5-fluorouracil, or 1,3-bis(2-chloroethyl)-1-nitrosourea. *J. Natl Cancer Inst.*, **39**, 257.

CARR, I. & CARR, J. (1981). Experimental lymphatic invasion and metastasis. In *Lymphatic System Metastasis,* Weiss, L. *et al.* (eds) p. 41. G.K. Hall: Boston.

CARTER, C.J., SPEAKMAN, H. & DIXON, B. (1980). The nuclear DNA content of a transplantable rat tumour: Comparison of primary growths and metastases. In *Flow Cytometry IV*, Laerum, O.D. *et al.* (eds) p. 436. Universitetsferluget: Norway.

DE RUITER, J., CRAMER, S.J., LELIEVELD, P. & VAN PUTTEN, L.M. (1982). Comparison of metastatic disease after local tumour treatment with radiotherapy or surgery in various tumour models. *Eur. J. Cancer Clin. Oncol.*, **18**, 281.

DIXON, B. & BAGNALL, D.A. (1985). Metastasis and the excision of irradiated $LMC_1$ tumours in the rat. *Radiol. and Oncol.*, **4**, 153.

DIXON, B. & BAGNALL, D.A. (1986). The pattern and timing of lymphatic metastasis of the rat carcinoma $LMC_1$. *Clin. Expl. Metastasis*, **4**, 117.

DIXON, B. & SPEAKMAN, H. (1979). Local recurrence and metastasis of excised breast carcinomas in the rat. *J. Roy. Soc. Med.*, **2**, 572.

FALK, P. (1980). The vascular pattern of the spontaneous C3H mouse mammary carcinoma and its significance in radiation response and in hyperthermia. *Eur. J. Cancer*, **16**, 203.

FIDLER, I.J. & HART, T.R. (1982). Host immunity in experimental metastasis. In *Immunological Aspects of Cancer,* Castro, J.E. (ed) p. 183. M.T.P.: London.

FIDLER, I.J. & POSTE, G. (1982). The heterogeneity of metastatic properties in malignant tumour cells and regulation of the metastatic phenotype. In *Tumour Cell Heterogeneity*, Owens, A. *et al.* (eds) p. 127. Academic Press: New York.

FOLKMAN, J. & TYLER, K. (1977). Tumour angiogenesis: Its possible role in metastasis and invasion. In *Cancer Invasion and Metastasis: Biological Mechanisms and Therapy*, Day, S.B. *et al.* (eds) p. 95. Raven Press: New York.

HEWITT, H.B. (1958). Studies of the dissemination and quantitative transplantation of a lymphocytic leukaemia of CBA mice. *Br. J. Cancer*, **12**, 378.

HEWITT, H.B. & BLAKE, E.R. (1975). Quantitative studies of the translymphnodal passage of tumour cells naturally disseminated from a non-immunogenic murine squamous carcinoma. *Br. J. Cancer*, **31**, 25.

HEWITT, H.B. & BLAKE, E.R. (1977). Further studies of the relationship between lymphatic dissemination and lymphnodal metastasis in non-immunogenic murine tumours. *Br. J. Cancer*, **35**, 415.

HEWITT, H.B., BLAKE, E. & PORTER, E.H. (1973). The effect of lethally irradiated cells on the transplantability of murine tumours. *Br. J. Cancer*, **28**, 123.

KIM, V. (1984). Metastatic patterns and properties of tumour cells. *Trans. Proc.*, **16**, 373.

LIOTTA, L.A., KLEINERMAN, J. & SAIDEL, G.M. (1976). The significance of haematogeneous tumour cell clumps in the metastatic process. *Cancer Res.*, **36**, 889.

MOORE, J.V. & DIXON, B. (1977*a*). Serial transplantation, histology and cellular kinetics of a rat adenocarcinoma. *Cell Tissue Kinet.*, **10**, 503.

MOORE, J.V. & DIXON, B. (1977*b*). Metastasis of a transplantable mammary tumour in rats treated with cyclophosphamide and/or irradiation. *Br. J. Cancer*, **36**, 221.

OWENS, A., COFFEY, D.S. & BAYLIN, S.B. (1982). *Tumour Cell Heterogeneity: Origins and Implications*. Academic Press: New York.

PETERS, L.J. & HEWITT, H.B. (1974). The influence of fibrin formation on the transplantability of murine tumour cells: Implications for the mechanism of the Révész Effect. *Br. J. Cancer*, **29**, 279.

PORTER, E.H., HEWITT, H.B. & BLAKE, E.R. (1973). The transplantation kinetics of tumour cells. *Br. J. Cancer*, **27**, 55.

RÉVÉSZ, L. (1956). Effect of tumour cells killed by X-rays upon the growth of admixed variable cells. *Nature*, **178**, 1391.

RÉVÉSZ, L. (1958). Effect of lethally damaged tumour cells upon the development of admixed viable cells. *J. Natl Cancer Inst.*, **20**, 1157.

SALMON, S.E. (1977). Kinetic rationale for adjuvant chemotherapy of cancer. In *Adjuvant Therapy of Cancer*, Salmon, S.E. & Jones, S.E. (eds) p. 15. North-Holland: Amsterdam.

SPEAKMAN, H. (1986). Metastasis of the $LMC_1$ rodent tumour and the response to treatment with cyclophosphamide in combination with surgery. *PhD Thesis*, University of Leeds.

SPEAKMAN, H. & DIXON, B. (1980). Quantitation and cellular growth of occult metastasis of a rat mammary carcinoma. In *Metastasis: Clinical and Experimental Aspects*, Hellman, K. *et al.* (eds) p. 55. Martinus Nijhoff: The Hague.

SPEAKMAN, H. & DIXON, B. (1981). An experimental approach to chemotherapy for occult metastases: A quantitative model. *Eur. J. Cancer*, **17**, 1287.

STEEL, G.G. (1977). *Growth Kinetics of Tumours*. Clarendon Press: Oxford.

VANDENRIS, M., DUMONT, P., SEMAL, P., HEIMANN, R. & ATASSI, G. (1985). Investigation of a new murine model of regional lymph node metastasis: Characteristic of the model and applications. *Clin. Exptl. Metast.*, **3**, 7.

VAN DE VELDE, C.J.H., VAN PUTTEN, L.M. & ZWAVELING, A. (1977). A new metastasizing mammary carcinoma model in mice: Model characteristics and applications. *Eur. J. Cancer*, **13**, 555.

WEISS, L. (1985). *Principles of Metastases*. Academic Press: London.

WONDERGEM, J., HAVEMAN, J. & VAN DER SCHUEREN, E. (1985). Influence of thorax irradiation on the survival of mice with spontaneous or artificial lung metastases from a transplantable mammary adenocarcinoma. *Int. J. Radiat. Oncol. Biol. Phys.*, **11**, 1127.